# Glucagon-like Peptide-1 Therapy in Obesity-Related Heart Failure with Preserved Ejection Fraction: Mechanisms, Clinical Evidence, and Implications

**DOI:** 10.3390/jcm15103767

**Published:** 2026-05-14

**Authors:** Malak Moones Abedi, Ibrahim Alabid, Wasim I. I. Alghoul, Rasha Aziz Attia Salama, Karmal Abdulkareem Hammood Al Lami, Hadil Marzougui, Batool Raed Rafiq Swalha, Safwan Ahmed Shaikh, Shehla Shafi Khan, Nahla Abd Elhadi Mohamed

**Affiliations:** 1 RAK College of Medicine, Ras Al Khaimah Medical and Health Sciences University (RAKMHSU), Ras Al Khaimah 11172, United Arab Emirates; malaek.21901100@rakmhsu.ac.ae (M.M.A.); wasim.21901111@rakmhsu.ac.ae (W.I.I.A.); karmel.21901077@rakmhsu.ac.ae (K.A.H.A.L.); batool.21901016@rakmhsu.ac.ae (B.R.R.S.); safwan.21901109@rakmhsu.ac.ae (S.A.S.); 2 Department of Community Medicine, RAK College of Medicine, Ras Al Khaimah Medical and Health Sciences University (RAKMHSU), Ras Al Khaimah 11172, United Arab Emirates; 3 College of Medicine, University of Sharjah, Sharjah 27272, United Arab Emirates; u22100302@sharjah.ac.ae; 4 Department of Psychiatry, RAK College of Medicine, Ras Al Khaimah Medical and Health Sciences University (RAKMHSU), Ras Al Khaimah 11172, United Arab Emirates; shehla@rakmhsu.ac.ae; 5 Department of Microbiology, RAK College of Medicine, Ras Al Khaimah Medical and Health Sciences University (RAKMHSU), Ras Al Khaimah 11172, United Arab Emirates; nahla@rakmhsu.ac.ae

**Keywords:** obesity-related HFpEF, GLP-1 receptor agonists, semaglutide, tirzepatide, phenotype-directed therapy

## Abstract

**Background:** Glucagon-like peptide-1 (GLP-1)-based therapies offer significant cardiometabolic benefits. Obesity-related heart failure with preserved ejection fraction (HFpEF) arises from a complex interplay of increased lipids, chronic inflammation, and metabolic disturbances. These factors not only exacerbate the disease but also affect GLP-1 pathways, supporting the potential role of GLP-1-based therapies in targeting this condition. **Objective:** This review aimed to synthesize the current evidence on GLP-1-based therapy in HFpEF, focusing on mechanisms of action, clinical outcomes, and practical significance. **Methodology:** A narrative review using PubMed and Scopus was conducted, including studies published between January 2020 and March 2026. Evidence from randomized trials, pooled analyses, mechanistic studies, and observational data was incorporated. **Results:** GLP-1-based therapies, including semaglutide and tirzepatide, demonstrated significant improvements in symptoms, exercise capacity, and quality of life. These benefits are closely linked to weight loss, reduced inflammation, and improved congestion indices. Tirzepatide use has also been associated with a reduction in heart failure-related complications. The underlying mechanisms likely involve coordinated effects on metabolism, inflammation, hemodynamics, and cardiac remodeling. Current evidence suggests that its efficacy in improving morbidity rates is stronger than its efficacy in reducing mortality rates. **Conclusions:** GLP-1-based therapies offer a promising, phenotypically targeted approach to managing obesity-associated HFpEF. However, their long-term effects on mortality remain unclear, highlighting the need for further research. Further studies should refine patient selection and define optimal clinical integration.

## 1. Introduction

Heart failure with ejection fraction preservation (HFpEF) has become an increasingly important component of the global heart failure burden. However, effective treatment remains a challenge because HFpEF is not a single disease entity, but rather a heterogeneous syndrome shaped by multiple co-morbid factors [1,2]. Among these factors, obesity has emerged as one of the most important clinically and mechanistically contributing factors. Recent data confirm that obesity-related HFpEF is not simply HFpEF occurring in overweight patients, but a distinct cardiometabolic phenotype characterized by increased symptom severity, greater exercise intolerance, impaired adipose tissue function, and a hemodynamic profile marked by reduced cardiac reserve and increased susceptibility to congestion [3,4].

This phenotype is increasingly biologically consistent. In patients with HFpEF, obesity-related biomarkers, such as leptin and C-reactive protein, are associated with the presence of the disease and reduced exercise capacity, highlighting the role of obesity-induced inflammation in clinical presentation [3]. Furthermore, supra-pericardial adipose tissue is associated with circulatory disturbances, reduced peak oxygen consumption, and worsening outcomes, confirming that obesity in HFpEF is a potent pathophysiological factor and not merely a concomitant disease [1,4]. These characteristics help explain why conventional heart failure therapies often achieve only modest gains in symptoms and functional capacity in patients with HFpEF, leaving a substantial residual burden of limitations and frequently leading to heart failure relapses [5].

In this context, GLP-1-based therapy has garnered significant attention as a potential therapeutic strategy targeting the disease phenotype. Initially developed for hyperglycemia and obesity, GLP-1 receptor activation has demonstrated broader significance in cardiovascular health in patients with obesity and cardiovascular and metabolic diseases, including a reduction in major cardiovascular complications with semaglutide use in overweight or obese patients with known cardiovascular disease [6,7,8]. These findings provided an important clinical basis but did not directly establish its role in the treatment of HFpEF. This changed with the emergence of specialized clinical trials for obesity-related HFpEF, which demonstrated that semaglutide improved heart failure symptoms, physical limitations, body weight, exercise capacity, and markers of inflammation in obese patients with HFpEF, regardless of whether they had type 2 diabetes [9,10]. Subsequent meta-analyses have reinforced the consistency of these effects and suggested a potential benefit in improving heart failure outcomes in patients with a slight or preserved reduction in ejection fraction [11,12].

More recently, tirzepatide has expanded the scope of this therapeutic effect by reducing the risk of cardiovascular death or heart failure exacerbation, primarily by reducing the incidence of heart failure exacerbations, while simultaneously improving the health status of obese patients with HFpEF [13]. The results of the SUMMIT program indicate that the observed benefits may extend beyond weight loss, encompassing positive effects on blood volume and pressure, systemic inflammation, and cardiovascular and renal injury [13,14]. Based on these data, the clinical focus has shifted from simply reducing body weight to targeting the key pathophysiological factors underlying the HFpEF pattern associated with obesity.

Despite this growing body of evidence, published studies remain scattered across HFpEF studies, obesity trials, pooled analyses, and mechanistic sub-studies. To date, no comprehensive review has focused on GLP-1 therapy within the biologically coherent phenotype of obesity-associated HFpEF, where interrelated mechanisms such as obesity, inflammation, congestion, and metabolic dysfunction collectively contribute to disease progression and treatment response.

Accordingly, this review explores the current evidence supporting GLP-1-based therapy in obesity-associated HFpEF, with particular emphasis on the rationale for the mechanism, recent clinical trial data, and whether the observed benefits extend beyond weight loss alone. By framing GLP-1-based therapy within a phenotypic context rather than considering it as a standalone pharmacological intervention, this review seeks to clarify its current clinical role in the management of obesity-associated HFpEF and to highlight the key questions that remain before its role can be fully defined.

## 2. Literature Search Strategy

This study was conducted as a narrative review designed to comprehensively synthesize the evolving evidence supporting GLP-1-based therapy in HFpEF, with a particular focus on phenotype definition, biological rationale, clinical trial evidence, and mechanisms extending beyond weight loss alone. A structured literature search was performed using PubMed and Scopus to identify relevant studies published between January 2020 and March 2026.

The search strategy combined controlled vocabulary and free-text terms with Boolean operators, including: “HFpEF,” “heart failure with preserved ejection fraction,” “obesity-related HFpEF,” “obese HFpEF,” “glucagon-like peptide-1 receptor agonist,” “GLP-1-based therapy,” “semaglutide,” “tirzepatide,” “incretin-based therapy,” “dual GIP/GLP-1 agonist,” “symptoms,” “exercise capacity,” “6-min walk distance,” “Kansas City Cardiomyopathy Questionnaire,” “NT-proBNP,” “congestion,” “diuretic use,” “inflammation,” “cardiac remodeling,” and “epicardial adipose tissue.” Additional targeted searches were conducted to identify pooled analyses, mechanistic sub-studies, expert consensus documents, and recent scientific statements relevant to phenotype-specific interpretation.

Peer-reviewed original research articles were prioritized, with a focus on randomized clinical trials, pooled analyses, mechanistic studies, cohort studies, and translational research directly related to obesity-related HFpEF and incretin-based therapy. High-impact reviews and expert guidance documents were also included when they added meaningful context or clinical insight. Studies not relevant to HFpEF, obesity-related cardiometabolic disease, or GLP-1-based therapy were excluded. Older landmark studies were included when needed to better frame the broader HFpEF phenotype.

Studies were selected based on relevance, methodological quality, and contribution to the conceptual and clinical understanding of phenotype-specific HFpEF. A total of 55 studies were included in the final synthesis, comprising randomized clinical trials, pooled analyses, mechanistic studies, observational research, and key expert consensus documents. The majority of included studies originated from North America and Europe, reflecting the geographical distribution of major clinical trials in this field. No formal statistical synthesis was performed; findings were qualitatively integrated to provide a clinically meaningful, phenotype-oriented interpretation of the available evidence.

Due to the large number of included studies, Table 1 summarizes key landmark clinical trials, while the full evidence base is described narratively.

## 3. Preserved Systolic Heart Failure Associated with Obesity as a Distinctive Cardiac Metabolic Phenotype and Therapeutic Target

Understanding obesity-associated HFpEF as a distinct cardiac metabolic phenotype is essential to explaining the growing role of GLP-1-based therapy as a phenotype-targeted treatment strategy. Rather than simply reflecting the overlap between overweight and conventional HFpEF, obesity-associated HFpEF represents a unique cardiac metabolic phenotype. In contemporary HFpEF patient populations, overweight and obesity constitute a significant proportion of cases and are associated with increased symptom severity, reduced exercise capacity, and poorer health-related quality of life [3,15]. This distinction is important because it highlights that obesity is not merely a co-morbidity but a major factor in disease progression, functional impairment, and potentially treatment response in a significant subset of HFpEF patients [1,3].

The biological pattern of this phenotype is shaped by dysfunction in inflammatory and metabolic regulation [16]. In a mechanistic study of patients with preserved ejection fraction and chronic dyspnea who underwent invasive cardiopulmonary stress testing, elevated levels of leptin, resistin, C-reactive protein (CRP), and interleukin-6 (IL-6), adenoviral dysfunction, and insulin resistance were associated with decreased peak oxygen consumption. Both CRP and leptin were independently associated with an increased likelihood of developing HFpEF. Elevated CRP and IL-6 were also associated with decreased adverse event-free survival. These observations support the notion that exercise intolerance in obesity-related HFpEF results from activated inflammatory pathways and pathways derived from adipose tissue, rather than solely from body mass or poor physical fitness [3].

Epicardial adipose tissue is one of the most prominent structural markers of this phenotype. Increased adipose tissue has been associated with worsening hemodynamics, including increased right ventricular filling pressure and decreased peak oxygen consumption [5,17]. Separate observational studies indicate the importance of both qualitative and quantitative factors: decreased adipose tissue density is associated with worsening cardiac metabolic status and an increased likelihood of complications, while increased adipose tissue volume predicts an increased risk of all-cause death and hospitalization for heart failure in patients with mildly reduced or maintained ejection fraction. Based on these findings, these results suggest that adipose tissue in HFpEF is biologically active and clinically significant, and not merely a transient symptom [18,19,20].

The negative effects of obesity in HFpEF are not limited to the heart; they extend to other parts of the body. Peripheral body composition abnormalities appear to directly contribute to reduced exercise capacity and increased disease severity [21]. In patients with obesity-related HFpEF, improved skeletal muscle quality, as measured by phase angle, was associated with higher weight-adjusted absolute oxygen consumption (O_2_), as well as lower edema index and NT-proBNP levels [22]. Lifestyle intervention studies support this view: calorie restriction combined with aerobic exercise improves oxygen levels and quality of life, while resistance training improves muscle quality and strength, and subsequent fat mass recovery is associated with reduced exercise capacity [15,23]. These data confirm that the syndrome reflects a comprehensive central and peripheral phenotype, rather than simply an isolated cardiac dysfunction.

In general, obesity-associated HFpEF can be considered a syndrome resulting from the accumulation of biologically active fats, chronic low-grade inflammation, hemodynamic disturbances with a tendency toward congestion, and peripheral dysfunction [22,24]. This perspective is supported by studies showing that modern obesity-related markers are associated with worse outcomes in HFpEF, without clear evidence of a protective obesity paradox [24]. Understanding this phenomenon is important because it provides a framework for therapies that target more than just fluid balance or neurohormonal pathways. It also helps explain why symptoms persist and exercise capacity is reduced in patients with conventional heart failure therapies, particularly when obesity and systemic metabolic inflammation play a major role in disease progression [1].

## 4. Mechanisms of Clinical Benefit in Obesity-Related Heart Failure with Preserved Ejection Fraction

### 4.1. Adiposity and Metabolic Regulation

One of the key mechanisms underlying the benefit of GLP-1-based therapy in obesity-associated HFpEF is the reduction of excess fat, particularly visceral and pericardial fat, which are strongly associated with circulatory disturbances, reduced exercise capacity, and disease progression [19,20,25]. This lipid-targeted approach is supported by clinical trials demonstrating significant weight loss with tirzepatide use in obese individuals, regardless of whether they have type 2 diabetes [26,27]. In addition to weight loss, GLP-1 receptor activation improves metabolic regulation by enhancing insulin sensitivity and modulating adipokine signaling, thereby addressing the major metabolic disturbances that characterize this condition [3,10]. Taken together, these effects suggest that the therapy targets not only body weight but also the underlying metabolic dysfunction that causes the disease.

### 4.2. Inflammation and the Metabolic–Inflammatory Axis

Obesity-associated HFpEF is characterized by chronic low-grade inflammation, which contributes to myocardial stress, vascular dysfunction, and reduced exercise capacity [3,16]. GLP-1-based therapies have consistently demonstrated their ability to reduce inflammatory markers such as C-reactive protein, and this reduction is associated with improvements in cardiac stress biomarkers and functional capacity [14,28]. This supports the notion that targeting the metabolic-inflammatory axis is a key mechanism by which these therapies achieve clinical benefit.

Beyond reductions in circulating inflammatory markers, several biological mechanisms may explain the anti-inflammatory effects of GLP-1-based therapies. GLP-1 receptor activation has been shown to modulate adipose tissue function by reducing pro-inflammatory cytokine production (e.g., interleukin-6 and tumor necrosis factor-α) and improving adipokine balance, including decreased leptin and increased adiponectin signaling. In addition, improvements in insulin sensitivity and reductions in lipotoxicity may attenuate activation of inflammatory pathways within both vascular and myocardial tissues. Emerging evidence also suggests that GLP-1 signaling may exert direct effects on endothelial function and oxidative stress, leading to reduced vascular inflammation and improved microvascular integrity. Collectively, these mechanisms provide a plausible biological framework linking GLP-1 receptor activation to systemic and tissue-level reductions in inflammatory burden observed in obesity-related HFpEF.

### 4.3. Congestion and Hemodynamic Load

Congestion and volume–pressure overload are key factors in the pathophysiology of obesity-associated HFpEF. GLP-1-based therapy appears to improve these hemodynamic abnormalities, as reflected by reductions in natriuretic peptide levels, estimated blood volume, and blood pressure [14,29]. These changes are accompanied by decreased reliance on loop diuretics and improved symptom burden, suggesting a clinically meaningful impact on congestion biology beyond weight reduction alone [29,30].

### 4.4. Cardiac and Adipose Tissue Remodeling

Emerging mechanistic and imaging data indicate that GLP-1-based therapy may favorably influence structural remodeling. Observed changes include reductions in left ventricular mass, improvements in diastolic parameters, and decreases in pericardial adipose tissue [31,32]. Given the recognized role of epicardial fat and structural remodeling in HFpEF pathophysiology, these findings provide a mechanistic link between metabolic intervention and cardiac functional improvement [5,17].

### 4.5. Integrated Interpretation: Weight Loss and Phenotype-Directed Effects

The overall therapeutic effect of GLP-1-based therapy appears to reflect a combination of weight-mediated and weight-independent mechanisms. While greater weight loss is consistently associated with improved symptoms and exercise capacity [33], parallel improvements in inflammatory markers, congestion indices, and structural parameters suggest broader phenotype-directed effects [28,29,31]. These results indicate that GLP-1-based therapy affects the underlying cardiac metabolic and inflammatory processes that contribute to obesity-associated HFpEF, rather than simply promoting weight loss.

**Figure 1:**
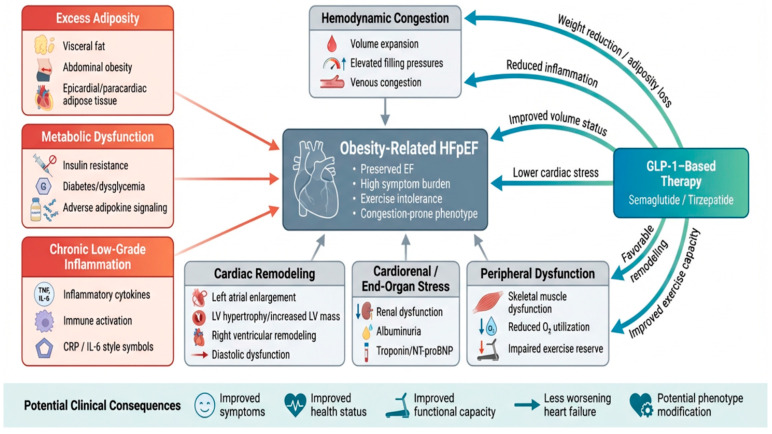
Pathophysiological basis of obesity-associated heart failure with preserved ejection fraction (HFpEF) and potential sites of action for glucagon-like peptide-1 (GLP-1)-based therapies. (Created using Bio Render, 2026).

In addition to its role as a clinical outcome, improved exercise capacity may itself represent a contributing mechanism underlying the broader benefits of GLP-1-based therapies. Enhancements in functional capacity are likely mediated by a combination of reduced adiposity, improved skeletal muscle quality, and decreased systemic inflammation, all of which contribute to more efficient peripheral oxygen utilization. Improved exercise tolerance may, in turn, facilitate increased physical activity, creating a positive feedback loop that further enhances metabolic regulation, cardiovascular efficiency, and overall functional status. This interaction between weight loss, physical performance, and systemic metabolic improvement highlights the importance of considering exercise capacity not only as an endpoint but also as a mediator of therapeutic benefit in obesity-related HFpEF. To connect these interrelated mechanisms, Figure 1 illustrates the pathophysiological basis of obesity-associated HFpEF and the primary targets of GLP-1-based therapy.

This figure illustrates obesity-associated HFpEF as a distinct cardiac metabolic phenotype, driven by increased lipids, metabolic dysfunction, chronic low-grade inflammation, circulatory congestion, cardiac remodeling, cardiorenal and peripheral organ stress, and limb dysfunction. It also shows the main pathways through which GLP-1-based therapies, including semaglutide and tirzepatide, may act beyond weight loss, such as reducing lipids, decreasing inflammatory signaling, improving fluid status, reducing cardiac stress, supporting cardiac remodeling, and enhancing exercise capacity. In general, these combined effects may translate into symptom improvement, improved health status, increased functional capacity, reduced heart failure exacerbations, and potential modification of the underlying disease phenotype. Abbreviations: HFpEF, heart failure with preserved ejection fraction; GLP-1, glucagon-like peptide-1; LV, left ventricle; NT-proBNP, amino-terminal B-type natriuretic peptide; CRP, C-reactive protein; O_2_, oxygen.

### 4.6. Integrated Mechanistic–Clinical Perspective

The clinical benefits observed with GLP-1-based therapies in obesity-related HFpEF appear to reflect the convergence of multiple interrelated biological pathways. Improvements in symptoms, functional capacity, and quality of life are closely aligned with reductions in systemic inflammation, congestion burden, and adverse cardiac remodeling. For example, reductions in inflammatory markers such as C-reactive protein have been associated with improvements in exercise capacity and health status, while decreases in estimated plasma volume and natriuretic peptide levels correspond with reduced congestion and symptom burden. Similarly, structural changes, including reductions in left ventricular mass and epicardial adipose tissue, provide a mechanistic basis for improved cardiac performance.

Overall, these findings suggest that GLP-1-based therapies exert integrated cardiometabolic and hemodynamic effects, linking mechanistic pathways to clinically meaningful outcomes rather than acting through weight loss alone.

## 5. Clinical Evidence of the Efficacy of GLP-1-Based Therapy in Treating Obesity-Related Heart Failure with Preserved Ejection Fraction

### 5.1. Semaglutide: STEP-HFpEF Program

Randomized data from the STEP-HFpEF program show that semaglutide significantly improves heart failure symptoms, physical limitations, and exercise capacity in patients with HFpEF associated with obesity but without diabetes, along with significant weight loss and reductions in inflammatory markers [9]. These benefits were reflected in improved Kansas City cardiomyopathy questionnaire scores and an increase in 6 min walking distance, highlighting clinically significant and patient-centered findings [34,35]. The effects were consistent across different obesity and baseline health status categories, suggesting broad applicability within this phenotype [33].

### 5.2. Semaglutide in Patients with Diabetes Mellitus (STEP-HFpEF DM)

The STEP-HFpEF DM trial extended these findings to include patients with type 2 diabetes, a group with more complex metabolic dysfunction. Despite a slight decrease in weight loss, improvements in symptoms, functional capacity, and inflammatory markers were similar to those observed in non-diabetic patients [10]. These results suggest that the benefits of semaglutide are not solely dependent on the degree of weight loss.

### 5.3. Pooled and Subgroup Analyses

Pooled analyses of semaglutide trials have shown consistent improvements in health status, exercise capacity, and body weight across a wide range of clinically relevant subgroups [11]. Larger pooled datasets, including trials such as SELECT and FLOW, also indicate a reduction in heart failure-related events, although clear evidence of the benefit of reduced cardiovascular mortality remains lacking [12]. Subgroup analyses have demonstrated consistent effects across sex, ejection fraction ranges (including mild and preserved reductions), and comorbidities such as atrial fibrillation, supporting the robustness and generalizability of these findings [36,37,38]. Further analyses suggest that patients with the more advanced NYHA functional class may experience greater improvements in their health status, despite achieving similar weight loss [39].

### 5.4. Tirzepatide: The SUMMIT Program

Recent evidence from the SUMMIT program indicates that tirzepatide reduces the risk of cardiovascular death or heart failure exacerbation, primarily due to a decrease in heart failure exacerbations, along with an improvement in the overall clinical course in patients with HFpEF and obesity [13,40]. Mechanistic analyses from the same program demonstrated reductions in blood pressure, estimated blood volume, markers of inflammation, and indicators of myocardial and renal injury, along with positive changes in cardiac structure and pericardial adipose tissue [14,32]. Benefits were also observed irrespective of diabetes status, supporting the view that obesity-related HFpEF rather than diabetes alone may be a key determinant of response [41]. These findings suggest that dual incretin-based therapy may extend benefits beyond symptom improvement to include meaningful reductions in heart failure morbidity.

### 5.5. Overall Interpretation of Clinical Evidence

**Figure 2:**
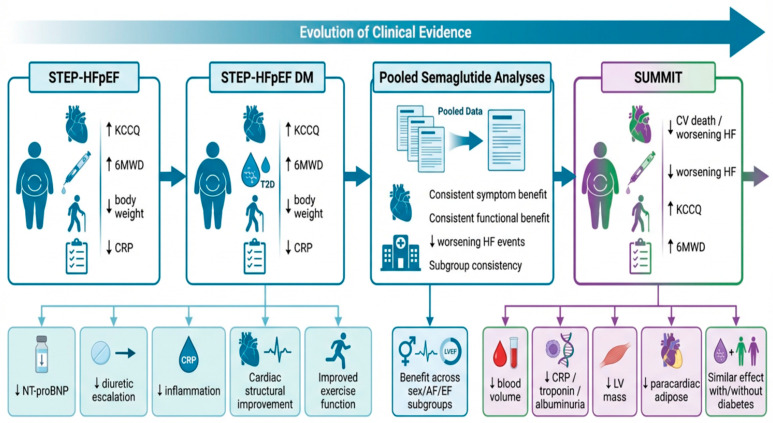
Clinical evidence for GLP-1-based therapy in obesity-related HFpEF. (Created in BioRender. Alghoul, W. (2026) https://BioRender.com/4rkwre8 (accessed on 7 May 2026)).

Taken together, the available clinical evidence demonstrates a high degree of consistency across therapeutic agents, study designs, and patient populations. GLP-1-based therapies, including semaglutide and tirzepatide, improve symptoms, functional capacity, and markers of congestion and inflammation, while also reducing heart failure-related events [9,10,11,12,13,14,29]. However, current evidence remains more robust for improvements in quality of life and morbidity than for definitive effects on long-term outcomes such as cardiovascular mortality [12,13]. This broader class-level signal is further supported by a 2025 systematic review and meta-analysis showing reductions in worsening heart failure events and in the composite of cardiovascular death or worsening heart failure, without a significant reduction in cardiovascular death alone [42]. The evolution and consistency of this clinical evidence are summarized in Figure 2.

This figure summarizes the evolution of the clinical evidence supporting GLP-1-based therapy in obesity-related HFpEF across STEP-HFpEF, STEP-HFpEF DM, pooled semaglutide analyses, and SUMMIT. Across these studies, treatment was associated with improvement in heart failure symptoms, health status, exercise capacity, and body weight, with additional mechanistic and clinical signals for lower inflammatory burden, reduced congestion-related burden, improved structural or cardiorenal markers, and fewer worsening heart failure events. The figure also emphasizes the growing consistency of benefit across clinically relevant subgroups and supports the concept that GLP-1-based therapy may provide broader phenotype-oriented benefit in obesity-related HFpEF beyond weight reduction alone. Abbreviations: HFpEF, heart failure with preserved ejection fraction; GLP-1, glucagon-like peptide-1; DM, diabetes mellitus; T2D, type 2 diabetes; KCCQ, Kansas City Cardiomyopathy Questionnaire; 6MWD, 6 min walk distance; NT-proBNP, N-terminal pro-B-type natriuretic peptide; CRP, C-reactive protein; LV, left ventricular; EF, ejection fraction; AF, atrial fibrillation; CV, cardiovascular.

Table 1 summarizes the key landmark clinical trials evaluating GLP-1-based therapies in obesity-related HFpEF.

## 6. Interpretation and Clinical Positioning

The current body of evidence suggests that glucagon-like peptide-1 (GLP-1)-based therapy represents a promising phenotype-directed approach in the management of obesity-related heart failure with preserved ejection fraction (HFpEF), a position also supported by recent focused reviews in this area [43]. The most consistent and clinically significant benefits are symptom relief, improved functional capacity, and increased health-related quality of life, along with reductions in congestion-related biomarkers and a decrease in heart failure [9,10,11,12,13,14,29]. However, these findings should be interpreted in light of current evidence, which remains stronger regarding disease-related outcomes than its apparent effects on cardiovascular mortality [12,13].

A key point to consider is the relative contribution of weight-related and non-weight-related effects. While greater weight loss is consistently associated with improved clinical outcomes [33,34], growing evidence suggests that additional mechanisms, such as reductions in inflammation, hemodynamic load, and cardiac remodeling, also play a significant role in overall therapeutic benefit [14,28,29,31]. This supports the view that GLP-1-based therapy provides integrated cardiac, metabolic, and hemodynamic effects, rather than being solely a weight-loss intervention. However, current evidence does not yet demonstrate that these therapies fully modify disease course in the traditional sense, particularly given the lack of reliable long-term mortality data. Significant questions also remain regarding long-term disease course modification, optimal patient selection, and the best ways to integrate these therapies into clinical practice [12,13,44].

Clinically, current evidence supports the use of GLP-1-based therapy in carefully selected patients with obesity-associated HFpEF, particularly those with severe symptoms, reduced exercise capacity, and a cardiac metabolic profile characterized by hyperlipidemia and metabolic disturbances [9,33]. These therapies should be used as adjuncts, not replacements, to established therapies, including sodium–glucose co-transporter-2 inhibitors, which remain the cornerstone of heart failure with preserved ejection fraction management [45,46]. Emerging clinical and observational data have also increased interest in the use of semaglutide and tirzepatide in patients with HFpEF, although these findings should be considered supportive rather than a change of practice [47,48]. The potential for complementary mechanisms of action among these therapies underscores the importance of a multimodal therapeutic approach that targets different aspects of the disease phenotype.

From a clinical perspective, GLP-1-based therapies should be considered adjunctive treatments within the current HFpEF management framework, rather than standalone interventions. Established therapies, particularly sodium–glucose cotransporter-2 (SGLT2) inhibitors, remain the cornerstone of HFpEF management. GLP-1-based therapies appear to provide complementary benefits by targeting obesity-related metabolic dysfunction, systemic inflammation, and congestion-related pathways.

Accordingly, their use may be most appropriate in selected patients with obesity-related HFpEF who remain symptomatic despite guideline-directed management, particularly those with prominent cardiometabolic features. However, optimal sequencing, combination strategies, and long-term impact on disease progression remain areas requiring further investigation.

At the same time, some important limitations in clinical practice must be considered. Most of the available evidence comes from studies conducted on select groups of stable outpatients, limiting its generalizability to patients with advanced disease, those whose condition has recently deteriorated, or those with serious comorbidities such as acute renal failure [25,49]. Furthermore, assessing drug tolerability (particularly gastrointestinal side effects) requires careful dose titration and continuous monitoring [50]. Consequently, current clinical use should focus on individualized patient selection, careful integration with existing therapies, and close monitoring of treatment response.

## 7. Clinical Implications and Practice Points

Patient selection: Glucagon-like peptide-1 (GLP-1)-based therapy appears to be most appropriate for patients with obesity-related HFpEF who have persistent symptoms, impaired exercise capacity, and a cardiac metabolic profile characterized by hyperlipidemia and metabolic disturbances, particularly in stable outpatient settings [9,10,33,51]. Current evidence does not support routine use in non-obesity-related heart failure with preserved ejection fraction or in patients with recently advanced or worsening disease [25,49].

Timing and Clinical Use: In clinical practice, GLP-1-based therapy should be considered for individuals who remain symptomatic despite guideline-directed management of HFpEF, with careful patient selection and gradual dose titration to improve tolerability and minimize gastrointestinal side effects [25,45,51].

Therapeutic positioning: These therapeutic agents are best suited as adjunctive therapies, not replacements for established treatments. Sodium-glucose cotransporter-2 inhibitors remain essential, while GLP-1-based therapies target complementary pathways associated with obesity, metabolic disorders, and inflammation [45,46]. Although recent evidence suggests potential additive or synergistic benefits with combination therapy, and comparative observational studies have reported positive outcomes for GLP-1 receptor agonists in diabetic HFpEF, optimal sequencing and integration strategies are still under investigation [25,46,52].

Monitoring and safety considerations: Patients receiving GLP-1-based therapy should be monitored for tolerability, hydration status, blood pressure, and nutritional adequacy, particularly in the elderly or frail individuals at risk of unintentional loss of lean body mass [23,25,51]. Concomitant therapies, including diuretics and glucose-lowering agents, may need to be adjusted [30].

Clinical Expectations: Clinically, these treatments are associated with improvements in symptoms, functional ability, and quality of life, along with a reduction in heart failure-related events, although current evidence is still more robust with respect to the reduction in morbidity than to the ultimate effects on cardiovascular mortality [9,10,11,12,13].

## 8. Future Directions

Several key priorities should guide the future development of GLP-1-based therapy in obesity-associated HFpEF. First, long-term outcome studies are needed to determine whether improvements in symptoms and morbidity rates translate into sustained benefits in cardiovascular disease progression and mortality [12,13]. While current findings are encouraging, they are limited by relatively short follow-up periods and a primary focus on functional and patient-reported outcomes.

Second, improving the classification of phenotypes is crucial. Body mass index alone is unlikely to adequately cover the complexity of obesity-related HFpEF. Future research should include more precise indicators, including measurements of visceral and pericardial fat, obesity-related anthropometric burden, inflammatory profiles, natriuretic peptide patterns, and imaging assessments of cardiac structure and function, to better identify patients most likely to benefit from treatment [20,29,53,54].

Third, the role of combination therapy requires further investigation. GLP-1-based drugs are likely to be used in conjunction with established treatments for heart failure that preserve contractile function, including sodium-glucose cotransporter-2 inhibitors and mineralocorticoid receptor antagonists. Randomized studies are needed to evaluate the cumulative or synergistic effects of these combinations, optimal sequencing strategies, and their impact on various clinical endpoints [25,45,46].

Fourth, mechanistic studies should continue to explore the relative contributions of weight loss and non-weight-related effects. Integrated approaches combining advanced imaging, biomarker identification, and functional assessments may help to clarify the extent to which these therapies modify underlying disease processes versus primarily improving clinical expression [31,33].

Finally, validating the findings on a larger scale in realistic populations is crucial. Future studies should include elderly patients, individuals with multiple comorbidities, and those with different phenotypes of HFpEF to assess the generalizability and practical applicability of the results [25,49]. Furthermore, factors such as long-term adherence, cost, accessibility, and tolerability will play a significant role in determining the integration of glucagon-like peptide-1 (GLP-1)-based therapy into routine clinical practice [51].

## 9. Limitations of Current Evidence

The findings presented in this review should be considered in light of several important limitations in the current evidence base. Most of the available data are derived from a relatively small number of randomized clinical trials conducted on selected groups of stable outpatients with obesity-associated HFpEF. Consequently, it remains unclear how applicable these findings are to broader and more diverse populations of patients with HFpEF, including those with advanced disease, recent deterioration, significant renal impairment, or non-obesity-related phenotypes [25,49].

Second, although sustained improvements in symptoms, functional ability, and health-related quality of life have been observed, the follow-up period in most trials remains relatively short. Consequently, the long-term sustainability of these benefits and their impact on key clinical outcomes, particularly cardiovascular mortality, have not yet been definitively established [12,13]. This limits the potential for classifying glucagon-like peptide-1 (GLP-1)-based therapies as fully disease-modifying interventions.

Third, a complete understanding of the relative contribution of weight-dependent versus non-weight-dependent mechanisms remains incomplete. While mechanistic analyses point to beneficial effects on inflammation, congestion, and cardiac remodeling, separating these effects from those mediated by weight loss is challenging and requires further research using integrated approaches based on physiology and imaging [29,31,33].

Fourth, variations in study design, patient selection, and outcome measures among trials may limit the possibility of direct comparison of results. Differences in baseline characteristics, presence of diabetes, degree of obesity, and previous heart failure treatments introduce potential variability in treatment response and interpretation of pooled results [9,10,11,12].

Finally, practical applicability in real-world settings remains an important consideration, especially since some comparative evidence is drawn from broader cohorts of heart failure and diabetes patients, rather than from obesity-associated HFpEF cohorts [55]. Issues related to long-term adherence, tolerability (particularly gastrointestinal side effects), cost, and accessibility may affect the efficacy of GLP-1-based therapies outside of controlled clinical trials [51]. Furthermore, current evidence provides only limited guidance on optimal integration with existing HFpEF therapies or on treatment sequencing in routine clinical practice.

These limitations, taken together, highlight the need for long-term studies with sufficient statistical power, improved phenotypic characterization, and realistic assessments to better define the role of GLP-1-based therapy in managing obesity-related heart failure while preserving contractile function.

## 10. Conclusions

Obesity-related heart failure with preserved ejection fraction (HFpEF) represents a distinct cardiometabolic phenotype characterized by the interplay of excess adiposity, systemic inflammation, congestion, and adverse cardiac remodeling. In this context, glucagon-like peptide-1 (GLP-1)-based therapies have emerged as a promising phenotype-directed approach in the management of this condition.

Current evidence suggests that GLP-1-based therapies, including semaglutide and tirzepatide, may improve symptoms, functional capacity, and health-related quality of life in patients with obesity-related HFpEF. These clinical benefits appear to be mediated through integrated mechanisms involving weight reduction, attenuation of inflammatory pathways, improvement in hemodynamic load, and favorable effects on cardiac and adipose tissue remodeling. However, these findings should be interpreted with caution, as the available evidence is more consistent for improvements in morbidity and patient-reported outcomes than for definitive reductions in cardiovascular mortality.

Importantly, GLP-1-based therapies should be viewed as adjunctive treatments within the broader HFpEF management framework, complementing established therapies such as sodium–glucose cotransporter-2 (SGLT2) inhibitors. Their role appears particularly relevant in selected patients with obesity-related HFpEF who remain symptomatic despite guideline-directed management and exhibit prominent cardiometabolic features.

Despite these encouraging findings, several uncertainties remain. The long-term impact of GLP-1-based therapies on disease progression and mortality, optimal patient selection, treatment sequencing, and integration with existing therapeutic strategies requires further investigation. In addition, variability in study populations and relatively short follow-up durations limit the generalizability of current evidence.

Overall, GLP-1-based therapy may represent an important step toward more personalized, phenotype-oriented management of HFpEF, although further high-quality, long-term studies are needed to define its role within routine clinical practice.

## Figures and Tables

**Table 1:** Landmark Clinical Trials of GLP-1-Based Therapy in Obesity-Related HFpEF.

Study	Population	Intervention	Design	Key Outcomes	Mechanistic Insights
STEP-HFpEF	Obese HFpEF	Semaglutide 2.4 mg weekly	RCT	↑ KCCQ, ↑ 6MWD, ↓ weight	↓ inflammation, ↓ congestion
STEP-HFpEF DM	HFpEF + T2D	Semaglutide 2.4 mg weekly	RCT	↑ symptoms, ↑ function	Benefits independent of diabetes
STEP pooled analyses	Mixed HFpEF	Semaglutide	Pooled RCTs	Consistent benefit across subgroups	Supports class effect
SELECT/FLOW analyses	CV risk patients	Semaglutide	Pooled	↓ HF events (trend)	Cardiometabolic improvement
SUMMIT	Obese HFpEF	Tirzepatide (up to 15 mg)	RCT	↓ HF exacerbations	↓ blood volume, ↓ inflammation
SUMMIT substudy	Obese HFpEF	Tirzepatide	Mechanistic	↓ LV mass, ↓ epicardial fat	Cardiac remodeling

Table 1 summarizes the major landmark clinical trials in this field. Additional supporting evidence, including mechanistic studies, observational research, and meta-analyses, is described in the text.

## Data Availability

No new data were created or analyzed in this study. Data sharing does not apply to this article.

## Abbreviations

The following abbreviations are used in this manuscript:

HFpEF	Heart failure with preserved ejection fraction
GLP-1	Glucagon-like peptide-1
GLP-1 RA	Glucagon-like peptide-1 receptor agonist
T2D	Type 2 diabetes
KCCQ	Kansas City Cardiomyopathy Questionnaire
6MWD	6 min walk distance
NT-proBNP	N-terminal pro-B-type natriuretic peptide
CRP	C-reactive protein
IL-6	Interleukin-6
LV	Left ventricular
EF	Ejection fraction
AF	Atrial fibrillation
SGLT2	Sodium-glucose cotransporter-2
BMI	Body mass index
CV	Cardiovascular
